# A novel visualization technique for measuring liquid diffusion coefficient based on asymmetric liquid-core cylindrical lens

**DOI:** 10.1038/srep28264

**Published:** 2016-06-21

**Authors:** Licun Sun, Xiaoyun Pu

**Affiliations:** 1 Department of Physics, Yunnan University, Kunming, Yunnan 650091, China

## Abstract

A visualization and quantification optical method for measuring binary liquid diffusion coefficient (*D*) based on an asymmetric liquid-core cylindrical lens (ALCL) is introduced in this paper. Four groups of control experiments were performed to verify the influences of diffusing substance category, concentration and temperature on diffusion process, and the measured *D* values were well consistent with data measured by Holographic interferometry and Taylor dispersion methods. The drifting of the diffusion image recorded by CCD reflects the diffusion rate visually in an easily understandable way. This optical method for measuring *D* values based on the ALCL is characterized by visual measurement, simplified device, and easy operation, which provides a new way for measuring liquid *D* value visually.

Diffusion is a process which leads to an equalization of concentrations within a single phase as a result of random molecular motions^1^. The study of diffusion is important in chemical engineering and other fields, such as biological systems, pollution control and separation of isotopes^2^. Diffusion coefficient (*D*) measurements provide fundamental information needed in various engineering and industrial operations, including the design of chemical reactors, liquid-liquid extractors, absorbers, distillation columns and so on^3^. In addition, the knowledge about diffusivity is also needed for the study of fluid-state theory, mass-transfer phenomena, and molecular interactions^4^.

Diffusion phenomena can appear in all gaseous, liquid and solid materials^1^,^5^. The color of a wall turns darker if a pile of coal is beside it for a long time in the absence of the external forces; the clear water becomes colored gradually when the iodine solution contacts with it without stirring; the scent of rose can be smelled even though there is no wind. Those are all diffusing phenomena, intuitive and easy to feel. Nevertheless, if two kinds of colorless transparent liquids are placed in contact, how can we present the diffusion process vividly and measure the *D* value accurately?

There are many well-established methods for the determination of liquid *D* value. Holographic interferometry^6^, diaphragm cell^7^ and Taylor dispersion^8^ are the most widely used techniques for diffusivity studies among them. Holographic interferometry method needs to analyze the interference fringes to work out the concentration profile of diffusive solutions^9^; diaphragm cell method requires to measure the initial and final concentrations of the upper and lower cell compartments^10^; Taylor dispersion method uses a refractive index detector to obtain a concentration profile that is similar with the Gaussian distribution curve^11^. Those three methods neither provide direct observation of the dynamic diffusion processes nor can be understood easily. Besides the common disadvantages mentioned above, interferometry method is time-consuming and requires extremely strict experimental environment^12^; the disadvantages of the diaphragm cell are also time-consuming and the necessity for calibration of the cell^13^; Taylor dispersion method makes use of a spiral capillary with length up to several meters and the mobile phase is required to flow through the round cross section at a constant flowing rate, which is difficult to achieve and leads to poor accuracy^14^.

To overcome these disadvantages, an asymmetric liquid-core cylindrical lens (ALCL), used as both diffusion pool and key imaging element, has been designed and used to measure liquid *D* values by our group recently^15^,^16^. ALCL can be used to measure refractive index (RI) of filled liquids in the way of spatial resolution along the axis of ALCL. If a dynamic gradient distribution of RI forms along the axis of ALCL because of molecule diffusion, ALCL projects a dynamic “beam waist” image on the CCD. The dynamic image reflects the diffusion process vividly, and the *D* value, based on the Fick’s second law, can be figured out either by recording the locations of “waist” at different times^15^ or by analyzing a simultaneous diffusion image rapidly^16^.

In this paper, we demonstrated the validity of our method to measure liquid *D* values through theoretical discussion and experimental results. The ALCL was utilized in the diffusion experiments of ethylene glycol-water (EG-water) and triethylene glycol-water (TEG-water) systems, the influences of solution concentration and temperature on diffusion were investigated, related *D* values were measured. Equipped with the ALCL and a CCD camera, all of diffusion processes can be visualized and displayed in the attached visualization files.

## Methods

### Imaging principle for ALCL filled with diffusion solution

**Figure 1:**
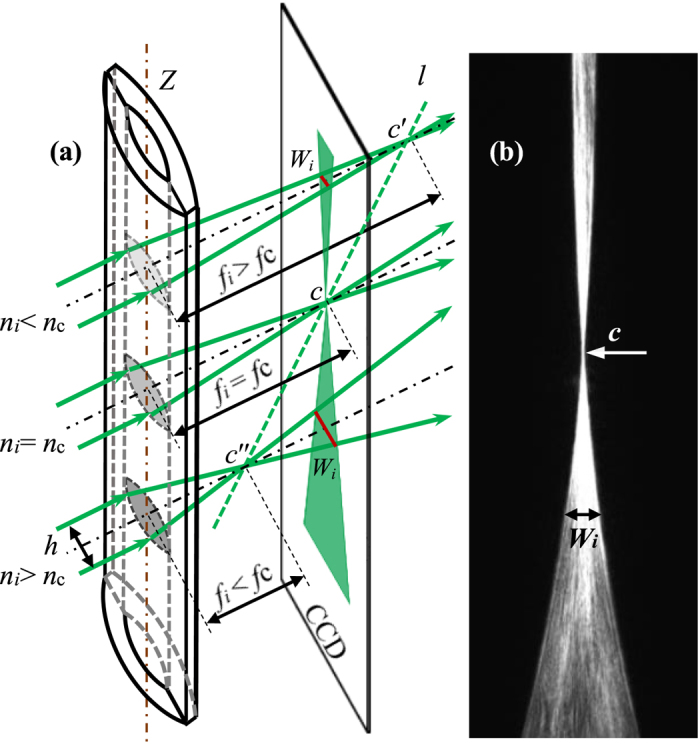
Imaging principle of ALCL. (**a**) a sloping focal curve (*l*) forms behind ALCL when a RI gradient distribution of the filled liquid is formed along *Z*-axis. (**b**) an experimental image recorded by CCD. The point *c* is the only clear imaging point relative to the thin liquid layer of *n*_*i*_ = *n*_*c*_, and *W*_*i*_ is the projected width when *n*_*i*_ is not equal to *n*_c_.

The imaging principle of ALCL filled with diffusion liquids is showed in Fig. 1(a). The iterative relations between the focal length *f*_*i*_ and RI of liquid (*n*_*i*_) filled in the ALCL can be written as,


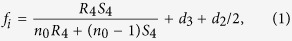



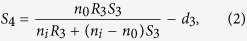



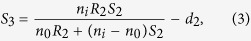



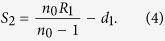


Here the curvature radii of the four refraction surfaces of ALCL are *R*_1_ = 20.0 mm, *R*_2_ = *R*_3_ = 17.0 mm, and *R*_4_ = 37.6 mm, respectively. The wall thicknesses and cavity thicknesses are *d*_1_ = *d*_2_ = *d*_3_ = 3 mm, respectively. The material of cylindrical lens is K9 glass (*n*_0_ = 1.5163 at λ = 589 nm). If parameter *f*_*i*_ is determined by measurement in advance, RI of liquid (*n*_*i*_) can be computed based on Eqs (1 ~ 4). The main purpose for designing the ALCL is to maintain a good RI measurement sensitivity and reduce spherical aberration to the minimum. The experimental setup and the method for measuring liquid RI based on the ALCL have been introduced in detail in refs 15 and 16.

Two kinds of diffusion solution are filled into the ALCL sequentially with the dense solution occupying the lower half. Once two solutions contact, the diffusion process commences. Dynamic gradient distributions of the concentration for the mixed solution are formed gradually along the diffusion direction defined as *Z*-axis. The focal length (*f*_i_) of ALCL for different thin liquid layer changes with the layer’s RI (*n*_*i*_). The collimation light will converge to be a sloping curve (*l*) after it passing through the ALCL filled with the diffusion solutions. If a CCD is fixed at the focal plane of the ALCL filled with liquid of RI = *n*_c_, as shown in Fig. 1(a), the curve *l* will only intersect with the image plane (CCD) at the point *c*, while the collimated beams passing the ALCL filled with liquid of RI = *n*_*i*_ > *n*_c_ (or *n*_*i*_ < *n*_c_) shall project a width *W*_*i*_ on the CCD plane. Figure 1(b) is the experimental image recorded by the CCD, consistent with the theoretical analysis. Let *h* be the width of collimated light beams, *f*_c_ and *f*_*i*_ be the focal length of the ALCL when filled with liquid of RI = *n*_c_ and RI = *n*_*i*_, respectively, from the view of geometrical optics, the image width (*W*_*i*_) and the focal length (*f*_*i*_) satisfy


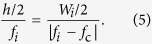


On the condition that *h* and *f*_c_ being known, *f*_*i*_ can be calculated by Eq. (5) when *W*_*i*_ is read out straightforwardly by a software built in the CCD camera. Inserting *f*_*i*_ to Eqs (1 ~ 4), *n*_*i*_, the RI of liquid filled in ALCL can be calculated. Since *W*_*i*_ value is varied with the *Z*-axis as shown in Fig. 1, *W*_*i*_ = *W*_*i*_(*Z*), after *f*_*i*_ = *f*_i_(*Z*) being determined by experimental method, *n*_*i*_ = *n*_*i*_(*Z*) and *C*_*i*_ = *C*_*i*_(*Z*), i.e., the spatial distribution of RI and concentration can be determined completely by only one diffusion image.

### Theoretical and computational methods for diffusion coefficients

Assuming binary solutions involved in diffusion to be A and B, diffusion direction to be *Z*-axis and *C*(*Z*, *t*) to be the mass fraction of A in B at diffusion time *t* and position *Z*, based on Fick’s second law, *C* (*Z*, *t*) satisfies the following equation^3^:


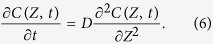


where, *D* is the diffusion coefficient. Assuming the initial concentrations to be *C*_1_ and *C*_2_ on each side of the contact interface (*Z* = 0) before the diffusion begins, according to the boundary and initial conditions, the solution of Eq. (6) is an error function that can be expressed as^17^


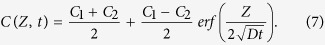


where, 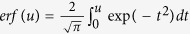 is the Gauss error function^18^. Equation (7) can be rewritten by the inverse error function as


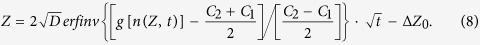


Here, 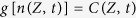 relating solution concentration to its RI, can be pre-determined experimentally. Δ*Z*_0_ is a fixed deviation of the position *Z* read out in actual measurements, as the contact interface cannot be an exact horizontal plane due to the intermolecular attraction. *C*_1_ is set to be 1, since pure diffusion chemical is filled in the lower part of ALCL, and *C*_2_ is a destination concentration filled on the upper part of ALCL.

The location (*Z*_*i*_) of a selected thin liquid layer, which has a fixed concentration and then a fixed RI = (*n*_c_), varies with diffusion time (*t*_*i*_), and the *D* values can be obtained by fitting linearly *Z*_*i*_ and 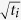
values and comparing it with Eq. (8), since 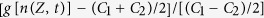 is a constant when the thin liquid layer is selected. The drift velocity of “waist” image reflects the diffusion rate intuitively.

## Results

To study the effect of diffusing substance category, concentration and temperature on diffusion process, four contrast experiments have been carried out, and the experimental contents are listed in Table 1.

**Figure 2:**
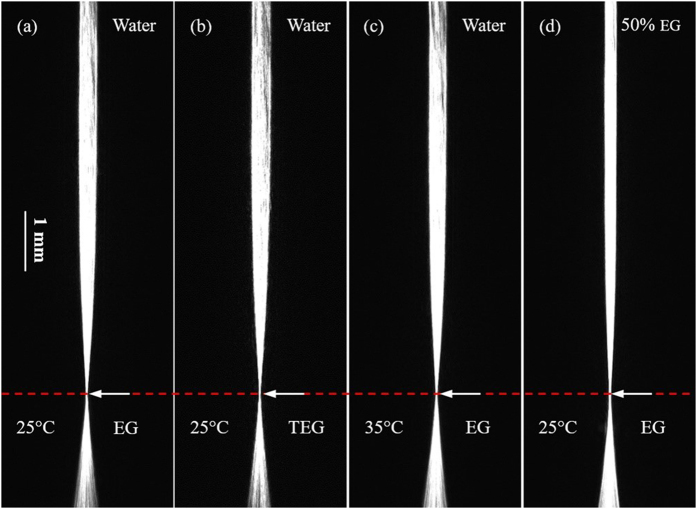
The images taken at the diffusion time of 10 minutes. (**a**) EG diffusing in pure water (*C*_1_ = 1, *C*_2_ = 0) at 25 °C. (**b**) TEG diffusing in pure water (*C*_1_ = 1, *C*_2_ = 0) at 25 °C. (**c**) EG diffusing in pure water (*C*_1_ = 1, *C*_2_ = 0) at 35 °C. (**d**) EG diffusing in 50% aqueous EG (*C*_1_ = 1, *C*_2_ = 0.5) at 25 °C. The arrows indicate the “waist” positions.

**Figure 3:**
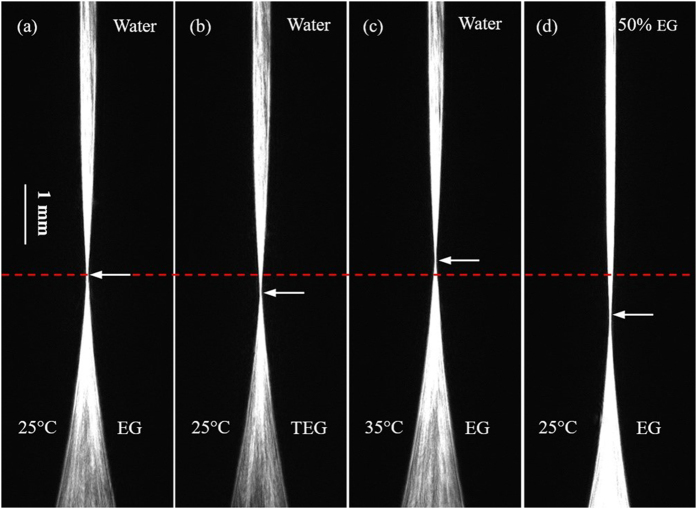
Same as Fig. 2, but the images were taken at the diffusion time of 45 minutes.

**Figure 4:**
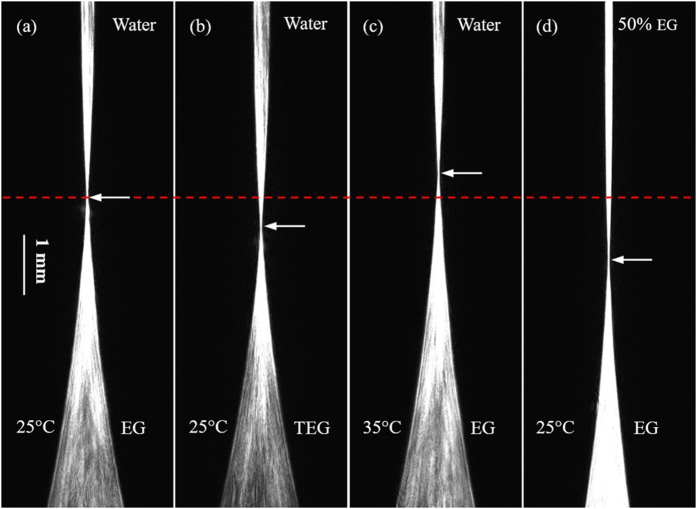
Same as Fig. 2, but the images were taken at the diffusion time of 90 minutes.

The experiment (a) is set as a contrast, and only one variable is changed in the other experiments. A CCD camera is regulated in a suitable vertical location, making the focus points (the “waist” of each image) of the four experiments at the same height in the initial recording time, as shown in Fig. 2(a). Dynamic concentration gradient distribution will form in the core area of ALCL due to the diffusion process, moreover, the focus point will drift with time as showed in Figs 3 and 4. The drift speed of the “waist” demonstrates the diffusion rate.

Comparing the experiment (b) with the experiment (a), it is clear that the larger molecule (MW_TEG_ = 150.18 > MW_EG_ = 62.07) has the larger friction among diffusion molecules, as a result, the larger molecule (TEG) has a lower diffusion rate as shown in Figs 3 and 4. The comparison between the two diffusion processes is displayed in the Supplementary Video S1.

Comparing the experiment (c) with the experiment (a), it is clear that higher temperature causes more vigorous random molecular motions, leading to a higher diffusion rate as shown in Figs 3 and 4. The comparison between two diffusion processes is displayed in the Supplementary Video S2.

Comparing the experiment (d) with the experiment (a), we can see that a dense diffusion solution gives rise to a short mean free path, leading to a low diffusion rate as shown in Figs 3 and 4. The comparison between two diffusion processes is displayed in the Supplementary Video S3.

In order to calculate *D* values, the “waist” locations varied with diffusion time in the four experiments are listed in Table 2, in which *N*_*i*_ means the number of pixel between the interface and the “waist” position and *Z*_*i*_ is equal to *N*_*i*_ multiplied by the pixel size (3.45 μm in this experiment). The RIs and mass fractions of the thin liquid layer (the “waist”), which are selected to image clearly on the CCD, are listed in the 2nd row and the 3rd row, respectively. The 4^th^ row shows the relationship between the RI and mass fraction. The 6^th^ to 16^th^ rows are experimental data recorded. The 17^th^ row indicates the fitting results between *Z*_*i*_ and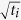. The *D* values can be calculated by comparing the fitting results with Eq. (8), which are listed in the 18^th^ row. The corresponding values in the previous work are listed in the 19^th^ row, which are well consistent with those we reported (the 18^th^ row).

## Discussion

Equation (6) is a fundamental differential equation of diffusion process, derived from the Fick’s first law, *F* = *D* ∂*C*/∂*Z*, and it works only when the *D* value is a constant. If *D* depends on the concentration of diffusing substance *C*, the Fick’s second law becomes^17^


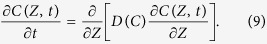


In this case, Eq. (8), the solution of Eq. (6), which is the base of our calculation by observing a thin liquid layer, is untenable. Therefore, the feasibility for calculating *D* value by Eq.(8) is determined mainly by whether the selected thin liquid layer meets the condition of *D*(*C*) ~ *D*(*C*_2_) in diffusion direction, where *C* and *C*_2_ are the concentrations of the thin liquid layer and the destination, respectively. Two experiments have been carried out to verify if the calculated *D* values are reasonable as follows.

**Figure 5:**
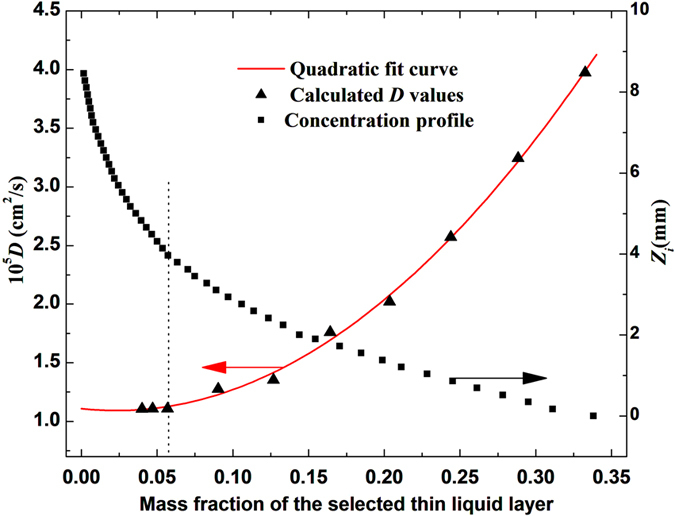
The calculated *D* values versus the measured concentration profile. Triangle dots: the calculated *D* values varied with selected thin liquid layers for EG diffusing in water (*C*_1_ = 1, *C*_2_ = 0). Square dots: concentration profile at the diffusion time of 45 minutes.

First, the destination concentration is set to be *C*_2_ = 0 (infinite dilution), thin liquid layers with different mass fractions have been selected to repeat the experiment (a), the calculated *D* values are presented by the triangle dots in Fig. 5. The spatial distributions of solution concentration at different diffusion time have been calculated by using Eq. (5) and the resulting diffusion images, the result at diffusion time of *t* = 45 minutes is shown in Fig. 5 by the squared dots. It is clear that the calculated *D* value is indeed dependent on the selected thin liquid layer, provided that when its mass fraction is larger than *C* = 0.057, corresponding to the area where concentration profile varies sharply with the diffusion distance, *D*(*C*) > *D*(*C*_2_ = 0). However, the measured *D* values tend to be a stable value of *D*(*C*_2_ = 0) = 1.1243 × 10^−5^ cm^2^/s when the selected mass fractions are near the destination *C* → *C*_2_ = 0, which corresponds to the area where concentration profile changes smoothly with the diffusion distance, *D*(*C*) ~ *D*(*C*_2_ = 0). Therefore, the stable value *D*(*C*_2_ = 0) = 1.1243 × 10^−5^ cm^2^/s is reliable in the condition of “infinite dilution”.

**Figure 6:**
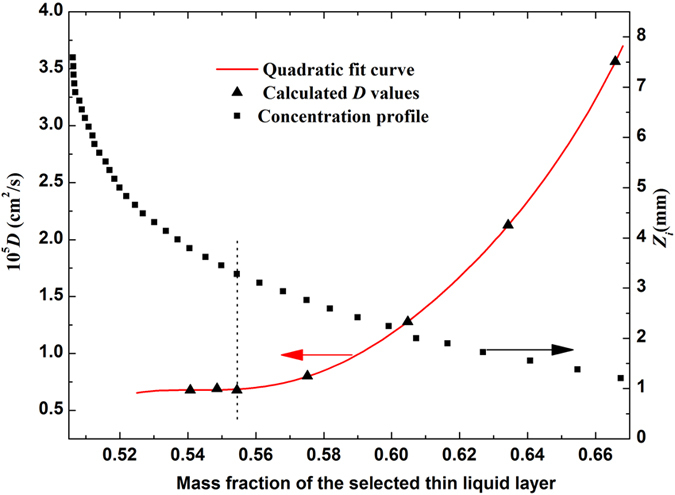
The calculated *D* values versus the measured concentration profile. Triangle dots: the calculated *D* values varied with selected thin liquid layers for EG diffusing in 50% aqueous EG (*C*_1_ = 1, *C*_2_ = 0.5). Square dots: concentration profile at the diffusion time of 45 minutes.

Second, the destination concentration is set to be *C*_2_ = 0.50, liquid layers with different mass fraction have been selected to repeat the experiment (d), and the calculated *D* values are presented by the triangle dots in Fig. 6. The spatial distributions of solution concentration at different diffusion time have been calculated by using Eq. (5) and the obtained diffusion images, and the result at diffusion time *t* = 45 minutes is shown in Fig. 6 by the squared dots. Figure 6 describes a similar situation as that in Fig. 5, the measured *D* values in Fig. 6 tend to be a stable value of *D* = 0.6768 × 10^−5^ cm^2^/s when the mass fractions of selected thin liquid layers are near the destination concentration *C*_2_ = 0.50, which is the *D* value of EG diffusing in the 50% aqueous EG.

In the method of Holographic interferometry, the concentration difference between two diffusion solutions, Δ*C* = (*C*_1_ − *C*_2_), is required to be small enough^19^ to render *D* value to be a constant; the Taylor dispersion method only injects a little bit of solute^20^ due to the same reason; there exists a conflict between differential *D* value measurement accuracy and its initial concentration difference across the diaphragm in classical diaphragm cell technique^21^. While, it is not necessary to maintain a small concentration difference in measuring *D* value accurately in the method invented by us, because it is fitted well by selecting a suitable thin liquid layer in diffusion cell, which reduces the instrumental requisition in measurement sensitivity. This is a new idea to measure the *D* values of different solution concentration. On the other hand, the concentration profile can be achieved conveniently based on Eq. (5) benefited from the large initial concentration difference, which appears to offer the potential for applying Boltzmann-Matano ideas^22^,^23^ to calculate liquid diffusivity of different concentrations, a usual method to measure *D* value varied with component concentration in solid diffusion, while never used in liquid to the best of our knowledge.

In summary, a new method to measure binary liquid *D* values based on an ALCL is presented in this paper. Four groups of contrast experiments have been carried out to verify the influence of diffusing substance category, concentration and temperature on diffusion process, and the measured *D* values are well consistent with data measured by Holographic interferometry and Taylor dispersion methods. The diffusion pool used in this method is more simplified compared with the Taylor dispersion method^20^, the measurement procedure is easier compared with the diaphragm cell technique^24^, and the required experimental environment is more relaxed than that needed in Holographic interferometry method^25^. Further more, this optical method for measuring liquid *D* value is characterized by visual measurement, short time consuming, and great potential for development, which paves a new way for visual measurements of liquid *D* values.

## Additional Information

**How to cite this article**: Sun, L. and Pu, X. A novel visualization technique for measuring liquid diffusion coefficient based on asymmetric liquid-core cylindrical lens. *Sci. Rep.* **6**, 28264; doi: 10.1038/srep28264 (2016).

## Supplementary Material

Supplementary Video S1

Supplementary Video S2

Supplementary Video S3

Supplementary Video Legends

## Figures and Tables

**Table 1:** Contents of four experiments.

Experiment (a)	Experiment (b)	Experiment (c)	Experiment (d)
EG diffusing in pure water at 25 °C.	TEG diffusing in pure water at 25 °C.	EG diffusing in pure water at 35 °C.	EG diffusing in 50% aqueous EG at 25 °C.
*C*_1_ = 1.0, *C*_2_ = 0	*C*_1_ = 1.0, *C*_2_ = 0	*C*_1_ = 1.0, *C*_2_ = 0	*C*_1_ = 1.0, *C*_2_ = 0.50.

**Table 2:** The data of position *Z*_*i*_ varied with diffusion time in the four experiments.

		Experiment (a)		Experiment (b)		Experiment (c)		Experiment (d)	
RI of liquid layer (*n*_c_)		1.3391		1.3397		1.3379		1.3898	
Mass fraction (%)		5.72		5.66		5.67		55.65	
*C* = *g*[*n*(*z*, *t*)]		9.8478*n*–13.130		7.9082*n*–10.538		10.0596*n–*13.402		9.8478*n*–13.130	
*t*_*i*_/s	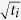	*N*_*i*_	*Z*_*i*_/μm	*N*_*i*_	*Z*_*i*_/μm	*N*_*i*_	*Z*_*i*_/μm	*N*_*i*_	*Z*_*i*_/μm
600	24.4949	548	1890.60	548	1890.60	548	1890.60	548	1890.60
900	30.0000	652	2249.40	643	2218.35	705	2432.25	616	2125.20
1200	34.6410	772	2663.40	744	2566.80	828	2856.60	694	2394.30
1500	38.7298	873	3011.85	815	2811.75	946	3263.70	747	2577.15
1800	42.4264	949	3274.05	883	3046.35	1035	3570.75	796	2746.20
2100	45.8258	1033	3563.85	940	3243.00	1118	3857.10	837	2887.65
2400	48.9898	1093	3770.85	988	3408.60	1183	4081.35	876	3022.20
2700	51.9615	1156	3988.20	1043	3598.35	1247	4302.15	907	3129.15
3000	54.7723	1202	4146.90	1080	3726.00	1325	4571.25	946	3263.70
3300	57.4456	1260	4347.00	1131	3901.95	1377	4750.65	978	3374.10
3600	60.0000	1307	4509.15	1168	4029.60	1424	4912.80	1006	3470.70
Fitting results between *Z*_*i*_ and 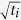		*Z*_*i*_ = 74.8676 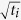 + 71.23 (μm)		*Z*_*i*_ = 60.2687 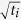 + 450.24 (μm)		*Z*_*i*_ = 84.8972 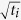 − 95.44 (μm)		*Z*_*i*_ = 44.5572 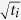 + 825.43 (μm)	
Calculated *D* values × 10^−5^ (cm^2^/s)		1.1243		0.7240		1.4386		0.6768	
*D* values in previous work × 10^−5^ (cm^2^/s)		1.102^26^		0.737^27^		1.381^26^		0.677^27^	
