# Supplementary material for: A novel visualization technique for measuring liquid diffusion coefficient based on asymmetric liquid-core cylindrical lens
Source: Sci Rep. 2016 Jun 21;6:28264. doi: 10.1038/srep28264 (PMC4915026; doi:10.1038/srep28264)
Supplement: Supplementary Video Legends [file srep28264-s4.doc]

**Supplementary Information**

**Manuscript title：** A novel visualization technique for measuring liquid diffusion coefficient based on asymmetric liquid-core cylindrical lens

**Author list：** Licun Sun and Xiaoyun Pu.

**Video legends:**

Video S1. The effect of diffusing substance category on diffusion process. The left of the video shows ethylene glycol-water (EG) diffusing in pure water (*C*_1_=1, *C*_2_=0) at 25°C, and the right of the video shows triethylene glycol-water (TEG) diffusing in pure water (*C*_1_=1, *C*_2_=0) at 25°C. Those two dynamic diffusion process showed in one video indicate that the larger molecule (MW_TEG_=150.18>MW_EG_=62.07) has the larger friction among diffusion molecules, as a result, the larger molecule (TEG) has a lower diffusion rate. Video S1 refers to the third paragraph from bottom of page 6 of the article file.

Video S2. The effect of temperature on diffusion process. The left of the video shows EG diffusing in pure water (*C*_1_=1, *C*_2_=0) at 25°C, and the right of the video shows EG diffusing in pure water (*C*_1_=1, *C*_2_=0) at 35°C. Those two dynamic diffusion process showed in one video indicate that higher temperature causes more vigorous random molecular motions, leading to a higher diffusion rate. Video S2 refers to the second paragraph from bottom of page 6 of the article file.

Video S3. The effect of diffusing substance concentration on diffusion process. The left of the video shows EG diffusing in pure water (*C*_1_=1, *C*_2_=0) at 25°C, and the right of the video shows EG diffusing in 50% aqueous EG (*C*_1_=1, *C*_2_=0.5) at 25°C. Those two dynamic diffusion process showed in one video indicate that dense diffusion solution gives rise to a short mean free path, leading to a low diffusion rate. Video S3 refers to the first paragraph from bottom of page 6 of the article file.
